# Comparative validation of a cost-effective in-house 3D-printed LLETZ simulator versus a commercial training model

**DOI:** 10.1007/s00404-026-08413-3

**Published:** 2026-04-04

**Authors:** Anne Cathrine Scherer-Quenzer, Katharina Reinhart, Joachim Diessner, Anastasia Altides, Johanna Buechel, Bettina Blau-Schneider, Stephanie Tina Sauer, Barbara Deschler-Baier, Achim Woeckel, Matthias Kiesel

**Affiliations:** 1 Department of Obstetrics and Gynecology, University Hospital of Wuerzburg, Josef-Schneider-Strasse 4, 97080 Würzburg, Germany; 2 Translational Oncology, Comprehensive Cancer Center Mainfranken, University Hospital Wuerzburg, Josef-Schneider-Str. 6, 97080 Würzburg, Germany; 3 Department of Radiology, University Hospital Freiburg, Hugstetter Str. 55, 79106 Freiburg, Germany

**Keywords:** 3D printing, LLETZ simulation, Surgical training, LEEP score, Video evaluation, Simulation training

## Abstract

**Purpose:**

Traditional surgical training methods such as "learning-by-doing" raise ethical and methodological concerns. To improve training and reduce patient risk, a 3D-printed LLETZ simulator was developed. While initial studies showed advantages over a conventional model, they were limited to medical students and lacked comparison with a commercially available simulator.

**Methods:**

A single-center study was conducted at the University Hospital Wuerzburg. 60 medical students without prior LLETZ experience and 10 gynecology residents with prior exposure were randomly assigned to train on either a commercial or the novel in-house simulator. Each participant performed five electrosurgical excisions. Performance was evaluated using LEEP scores, resection status (R0 resections), and blinded video assessments by two senior clinicians. Additionally, participants completed questionnaires, to capture subjective training impressions.

**Results:**

The in-house simulator demonstrated superior performance compared to the commercial model. When analyzed separately, changes in LEEP scores over the five attempts were statistically significant for both simulators. The effect size was larger for the in-house simulator (*η*^2^ = 0.227) than for the conventional simulator (*η*^2^ = 0.10). Within-group analysis revealed no significant pairwise differences across all attempts for the commercial simulator. In contrast, several pairwise comparisons remained statistically significant for the in-house simulator (attempts 1 vs. 3, 1 vs. 4, 2 vs. 5, and 1 vs. 5), all with large effect sizes (Cohen’s d > 1.1). Between-group comparison of individual LEEP scores showed a statistically significant difference in the fifth attempt (*p* = 0.002), with a large effect size (Cohen’s *d* = 1.01) favoring the in-house simulator. Higher R0 resection rates were observed with the in-house simulator in the third (100% vs. 83.3%) and fourth attempts (96.7% vs. 73.3%). Blinded video assessments by two senior experts confirmed these findings, demonstrating higher checklist, Global Rating Scale (GRS), and overall mean scores for the in-house simulator from the third attempt onward (all *p* < 0.05; all Cohen’s *d* > 0.8). Participant feedback further supported these results, indicating improved confidence, technical skills, and perceived educational value.

**Conclusion:**

This study demonstrated that the novel in-house 3D-printed simulator significantly outperformed the commercial model in objective surgical performance, learning progression and user satisfaction.

**Supplementary Information:**

The online version contains supplementary material available at 10.1007/s00404-026-08413-3.

## What does this study add to the clinical work

**Table:** 

This study demonstrates that an in-house 3D-printed LLETZ simulator provides significant advantages over a commercially available model for LLETZ training, leading to higher R0 resection rates and superior procedural quality. Clinically, improved early training may translate into safer procedures, fewer incomplete excisions, a reduced need for repeat interventions, and ultimately better patient outcomes in the management of cervical intraepithelial neoplasia.	

## Introduction

In 2020, approximately 4640 women in Germany were diagnosed with cervical cancer [1]. Worldwide cervical cancer was the fourth leading cause of cancer-related illness and death, with an estimated 662,044 new cases in 2022 [2]. As most cervical carcinomas develop from cervical dysplasia [3], a well-established national screening program aims to detect precancerous lesions at an early stage to prevent progression to invasive cancer. In cases of high-grade dysplasia, therapeutic excisional procedures, such as large loop excision of the transformation zone (LLETZ), are often indicated [3–6]. It is estimated that more than 100,000 such procedures are performed annually in Germany [7]. Although LLETZ is considered a routine gynecologic procedure, it carries the risk of several complications, which are directly related to the extent of the excision. While excessive tissue removal increases the risk of obstetric complications [8–14], an incomplete excision may elevate oncologic risk and necessitate resurgery [10, 12, 15, 16]. Further potential risks include acute vaginal injuries leading to hemorrhage, stenosis, fibrosis or even rectal injury [17–25]. Thus, surgical training plays a crucial role in minimizing potential complications [15, 26, 27].

Traditional surgical education often relies on a "learning-by-doing" approach, which is increasingly criticized due to both ethical and methodological concerns [28, 29]. As LLETZ procedures are frequently performed by gynecology residents, simulation models should be prioritized to mitigate the risks associated with inadequate experience. To address this need, Kiesel et al. developed a novel 3D-printed simulator designed to facilitate both the diagnosis of cervical dysplasia and the performance of LLETZ [30]. In a follow-up study, this model was compared to a conventional simulator which was designed based on the findings of Takacs et al. [31, 32]. While the in-house simulator demonstrated several advantages in training for the diagnosis and management of precancerous cervical lesions compared to the conventional simulator, the study had notable limitations. The simulator was assessed exclusively by medical students with no prior experience in performing electrosurgical excisions, thus limiting their capacity to accurately evaluate the realism of the simulator [30]. Moreover, the novel simulator was not compared to a commercially available model, which may have offered enhanced fidelity. To address these limitations, the presented study expands upon the previous evaluation by comparing the validated 3D-printed simulator with a commercially available model. Furthermore, the quality of the excision was not only assessed based on the LEEP score (loop electrosurgical excision procedure score) of the excised specimen [32], but also through a video-based analysis of surgical performance by two independent senior experts in gynecology and colposcopy. Finally, feedback from obstetrics and gynecology residents was incorporated, offering a more robust and clinically relevant evaluation of both simulators.

## Materials and methods

### Study design and study population

**Fig. 1:**
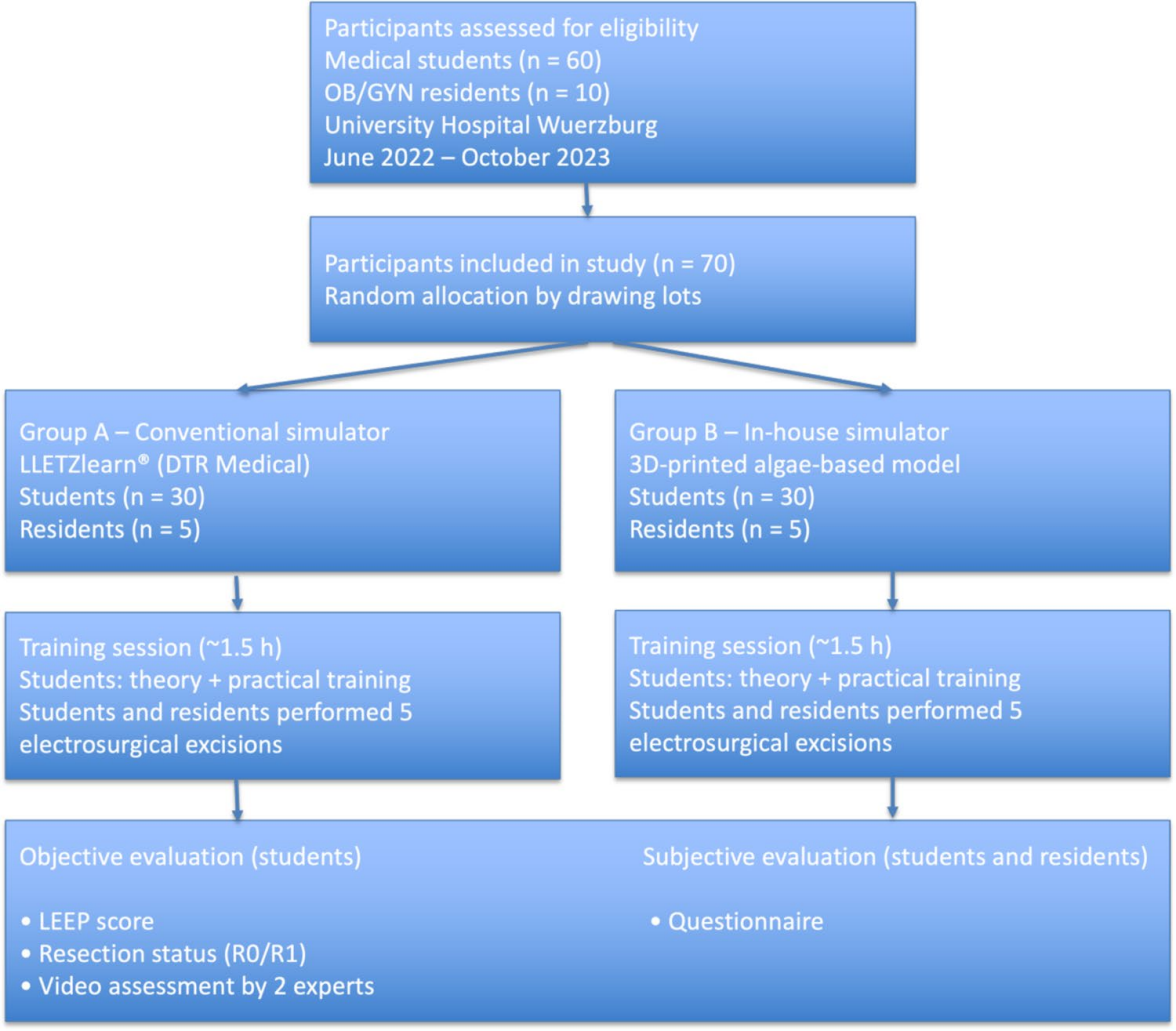
Flow chart of participant recruitment, allocation, and analysis. Medical students (*n* = 60) and gynecology residents (*n* = 10) were enrolled in the study and randomly assigned to either the conventional simulator (Group A) or the in-house developed simulator (Group B). Medical students and residents performed five electrosurgical excisions. Students were objectively evaluated using the LEEP score, resection status, and expert video assessment. Residents participated to provide subjective feedback regarding simulator realism and usability

This single center study was conducted at the Department of Gynecology and Obstetrics of the University Hospital Wuerzburg between June 2022 and October 2023. A certificate of non-objection was obtained from the Ethics Committee of the University Hospital Wuerzburg (application number 2020080401). The study population consisted of two cohorts. The first cohort included 60 medical students from Julius-Maximilians-University Wuerzburg, all of whom were in at least in the final third of their medical studies. None had prior experience performing LLETZ procedures. The second cohort consisted of 10 obstetrics and gynecology residents from the University Hospital Wuerzburg. In contrary to the medical students, these residents had prior hands-on experience in performing LLETZ. Participants (students and residents) were randomly assigned to either Group A or Group B by drawing a ticket from a box. Group A trained using the commercially available LLETZlearn^®^ Training Simulator from DTR Medical (DTR Medical Ltd, Swansea, UK), referred to as the conventional simulator. In accordance with the manufacturer’s guidelines and prior research, a sausage was utilized as a cervical substitute. Group B utilized the novel simulator, which had been introduced and validated in prior studies by Kiesel et al. [30, 31], referred to as the in-house simulator. As described by Kiesel et al., modified algae powder, 3D-printing and casting techniques were utilized to create this simulator. To ensure comparability between the two simulators, the commercial model was prepared using a standardized approach. The cervical component of the commercial simulator was created using a sausage segment that approximated the agar–agar cervix used in the in-house model. The segments of the sausage were shaped with a vegetable peeler into an identical conical form. Thus, the sausage cervix closely reassembled the geometry of the agar–agar cervix. The same preparation procedure and sausage type were used consistently for all training sessions to minimize variability between models. This approach allowed us to achieve a comparable cervical size and geometry between the commercial simulator and the in-house agar–agar model (Fig. 1).

### Organization and implementation of the seminar

Each training session lasted approximately 1.5 h and included a theoretical introduction delivered via Microsoft PowerPoint (Microsoft Corporation, Redmond, USA). The lecture provided an overview of essential topics, including female anatomy and physiology, the pathophysiology of cervical dysplasia, its diagnosis and treatment, and the principles, operation, and safety considerations of electrosurgery. For residents, who were already familiar with these concepts through their clinical experience, the theoretical introduction was omitted. Electrosurgical excisions were performed using the VIO 300D® electrosurgical generator, with neutral electrodes provided by the NESSY® Omega Plate (both from Erbe Elektromedizin GmbH, Tuebingen, Germany). Participants selected the appropriate electrosurgical loop following an inspection of both the simulator and the cervix, choosing from three available sizes (10 × 10 mm, 15 × 12 mm, and 20 × 15 mm; all loops were 130 mm long, Wolfram Schlingenelektrode, Erbe Elektromedizin GmbH, Tuebingen, Germany). Before their first attempt, participants were informed that the ideal depth of the excised cone should be between eight to ten millimeters. During the procedure, the instructor assisted in preparing the simulators, but did not provide surgical guidance. Following each excision, the depth of the cone was measured using a caliper and participants received immediate feedback, enabling them to determine, whether a further excision was necessary. The number of attempts and the corresponding cone depths were documented. Additionally, each excision process was recorded anonymously with the video camera of the colposcope only showing the loop and the cervix. After each procedure, the artificial cervix was replaced, and participants repeated the process until they had completed five electrosurgical excisions. The residents' simulations were neither evaluated using the LEEP score nor recorded on video. The primary objective for the residents was to gather their feedback, to assess the clinical acceptance of the simulator.

### Methods for objective evaluation

To evaluate the excised tissue, both the resection status and the LEEP score were assessed. The LEEP score is an objective evaluation method established by Takacs et al. [32, 33], which has been validated and applied by the aforementioned research group as well as in our previous studies [30, 31]. Each excision was scored based on the depth of the excised cone and the number of resections required, to achieve the desired depth. Each mm deviation of the optimal cone depth of 8–10 mm added one point. Each additionally performed resection also added one point. The optimal outcome was a score of zero points. In such a case, a cone depth of 8–10 mm was achieved with one single resection. Hence, higher scores indicated a greater deviation of the excised cone from the study’s ideal target and/or more excisions. The resection status (R-status) was also assessed, based on the first excision attempt. Each cervix-model showed a similarly marked area depicting the precancerous lesion using white color. If visible white residual tissue remained after the initial cut, the attempt was classified as R1. This classification remained unchanged even if subsequent corrective excisions completely removed the white tissue. Conversely, if the white tissue was entirely excised in the first attempt, the procedure was classified as R0. To assess surgical proficiency and the learning process, video evaluations were conducted by two independent senior experts in gynecology and colposcopy. The assessment was based on three scoring systems previously evaluated and applied by Wilson et al. [34]. The first scoring system, the checklist score, evaluated three key aspects: (1) whether the procedure was completed in a single step, (2) whether an adequate tissue sample was obtained, and (3) whether damage to the surrounding tissue was avoided. Each category was scored on a scale of 0–3 points, with a maximum possible total of 9 points. The second scoring system, the Global Rating Scale (GRS) score, assessed four criteria: (1) tissue handling, (2) time and motion efficiency, (3) instrument handling, and (4) procedural fluidity. Each category was rated on a scale from 1 to 5 points, with higher scores indicating superior performance. To calculate the overall score, the checklist score and the GRS score assigned by each expert were summed. For further analysis, the mean values of the checklist score (checklist mean), the GRS score (GRS mean) and the overall score (overall score mean) were determined across both evaluators.

### Methods for subjective evaluation

The second part of data collection aimed to assess the participants' subjective impressions. Both medical students and residents completed questionnaires at the end of the seminar. Responses were recorded using a 10-point Likert scale (1: "strongly agree/very good" to 10: "strongly disagree/very bad"). The questionnaire was adopted from the previous study by Kiesel et al., which was based on a validated questionnaire developed by Takacs et al. [31–33]. Depending on the participant group (students vs. residents, conventional simulator vs. novel simulator), certain questions varied between versions of the questionnaire.

### Statistical analysis

All variables were documented in an Excel file (Microsoft Office Excel 2013, Washington, USA). The data were analyzed using the statistical software SPSS (Version 29) from IBM (SPSS Inc., Chicago, IL. USA) and the following statistical test procedures:

- Descriptive Data Analysis.
- Friedman test and Mann–Whitney U test.
- p values were adjusted for multiple comparisons using the Holm–Bonferroni method.
- Effect sizes were calculated using eta squared (*η*^2^) for Friedman tests, Cohen’s d for pairwise comparisons and Cramer V for Fisher’s Exact test.
- The interrater reliability was calculated using Cronbach α.

### Use of large language models

ChatGPT Version 4.0 (OpenAI Inc., San Francisco, USA) was used for a language quality check.

## Results

The in-house simulator showed advantages over the conventional simulator.

### LEEP score and resection status

**Fig. 2:**
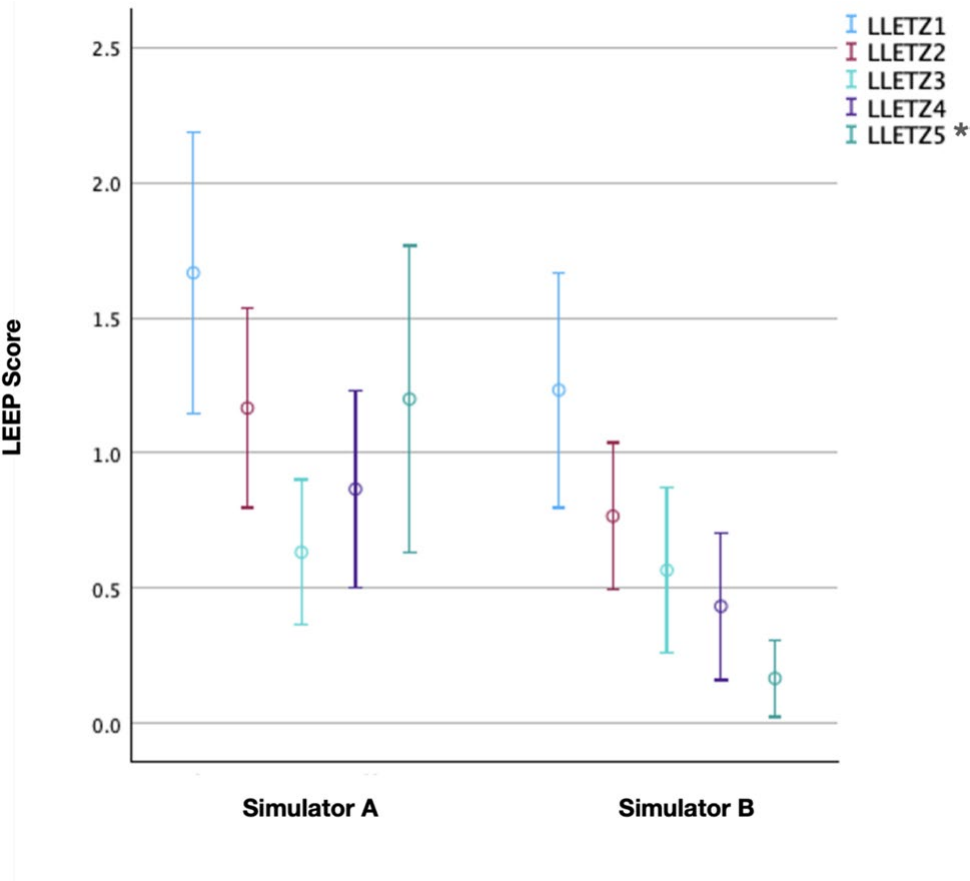
Mean LEEP score of simulator A and B over the course of five electrosurgical excisions. Dots represent the mean values. Vertical bars indicate the 95% confidence intervals (CI). Data points on the left correspond to simulator A (training with the conventional simulator), whereas those on the right represent simulator B (training with the in-house 3D-printed simulator). A statistically significant difference between both simulators was found in attempt five (green data points, adjusted *p* = 0.002). * indicates a statistically significant difference

Regarding the LEEP scores, the average of all recorded scores for LLETZ attempts one to five was calculated for both simulators. A LEEP score of zero points was regarded as the best possible result. In such a case, a cone depth of 8–10 mm was achieved with one single resection. Hence, higher scores indicated a greater deviation of the excised cone from the study’s ideal target and/or more excisions. The mean scores facilitated a more accurate comparison between the two phantoms. The mean scores for the conventional simulator exhibited a downward trend at the beginning, starting from an initial value of 1.667 in the first attempt, dropping to 1.167 in the second attempt and further decreasing to 0.633 in the third attempt. The LEEP scores then began to rise again: in the fourth attempt, the average LEEP score was 0.867, and in the fifth attempt, it increased to 1.200. The mean LEEP scores for the in-house simulator consistently decreased. The average score in the first attempt was 1.233, which dropped to 0.767 in the second attempt, 0.567 in the third, and 0.433 in the fourth attempt. Finally, in the fifth and last attempt, an average LEEP score of 0.167 was reached (Fig. 2). The comparison of the LEEP score results for the individual attempts between the two simulators was assessed using the Mann–Whitney-U-test. Effect size was calculated using Cohen’s d. A statistically significant difference between the two simulators was found in attempt five. The unadjusted *p* value was < 0.001 and the result remained significant after Holm adjustment (adjusted *p* = 0.002). The effect size was large, with a Cohen’s d of 1.01.

The variation in LEEP scores with the use of each simulator across the five attempts was assessed using the Friedman test. Effect size was calculated using eta squared (*η*^2^). A statistically significant change in LEEP scores over time was observed for both simulators. The corresponding effect size for the conventional simulator was *η*^2^ = 0.10, whereas the in-house simulator demonstrated a larger effect size (*η*^2^ = 0.227). Pairwise comparison within both groups were assessed using the Friedman test. Effect sizes for pairwise comparisons were calculated using Cohen’s d. After adjustment for multiple comparisons according to the Holm–Bonferroni method, no pairwise comparison remained statistically significant for the conventional simulator. In contrast, within the in-house simulator group, the differences between attempts 1 and 3, 1 and 4, 2 and 5, as well as 1 and 5 remained statistically significant following Holm correction and the corresponding effect sizes were large with all values exceeding 0.8 (Table 1).

**Table 1:** Pairwise comparison of LEEP scores across five attempts for both simulators

Comparison of two samples	Conv. simulator adjusted *p* value	Conv. Simulator Cohen’s *d*	In-house simulator adjusted *p* value	In-house simulator Cohen’s *d*
LLETZ1 – LLETZ3	0,060	1,176	0,042*	1,176
LLETZ1 – LLETZ4	0,333	0,822	0,027*	1,322
LLETZ1 – LLETZ5	0,553	0,677	< 0,001*	2,287
LLETZ 2 – LLETZ5	> 0,999	0,301	0,040*	1,199

* indicate statistically significant differences between the LEEP scores. Differences between the LEEP scores of other attempts were not statistically significant

**Fig. 3:**
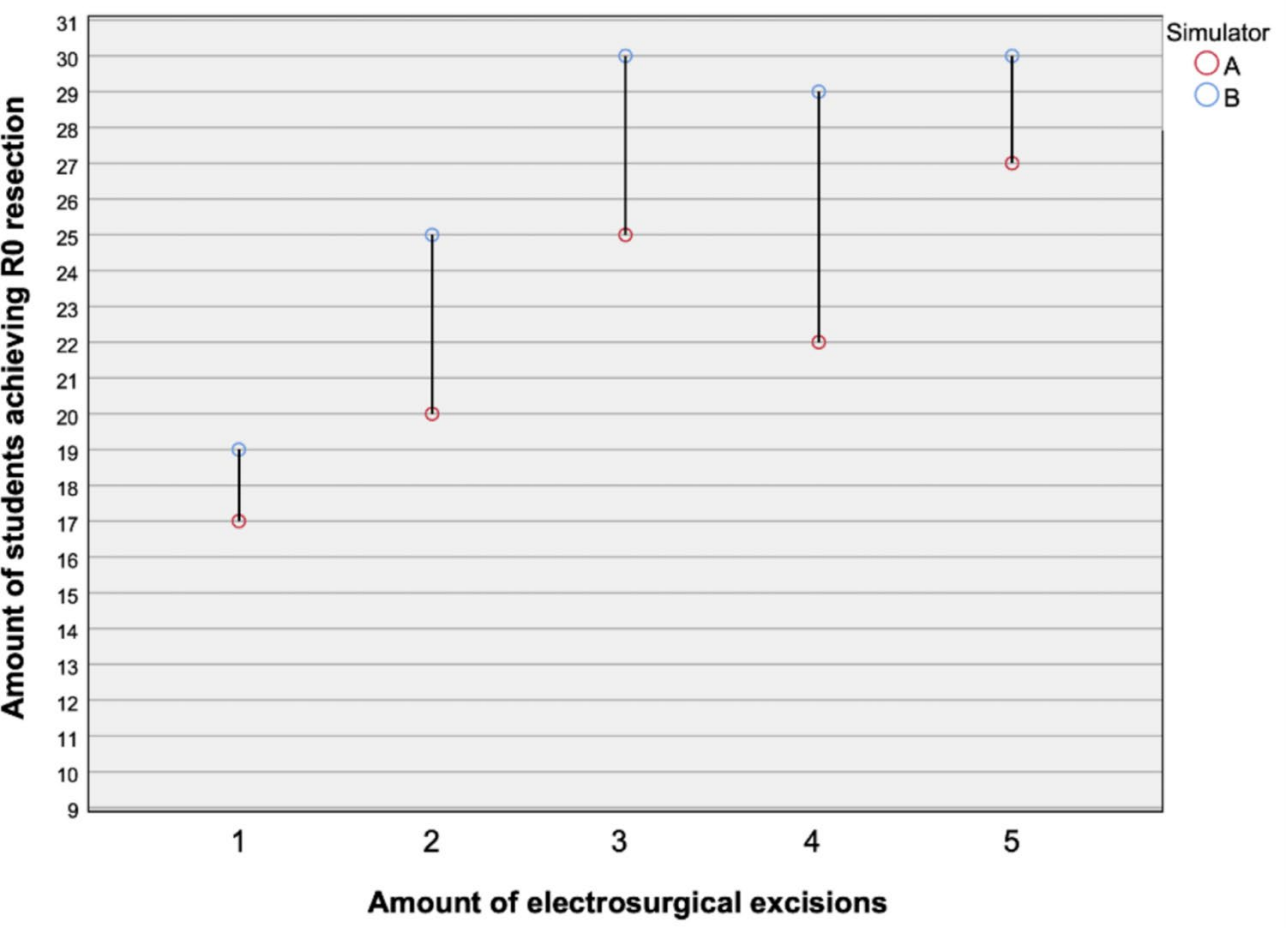
Amount of students achieving R0 resections over the course of five electrosurgical excisions. Red dots refer to simulator A (training with conventional simulator) and blue dots refer to simulator B (training with in-house simulator)

Regarding R0 resections, there was no statistically significant difference between the two simulators. During the first attempt, 17 students achieved complete (R0) resection using the conventional simulator (56.7%), and 19 participants (63.3%) using the in-house model. During the second attempt, 20 students (66.7%) in the conventional group reached R0 status, compared with 25 students (83.3%) in the in-house group. In the third attempt, all students (100%) achieved R0 resection with the in-house simulator, while only 25 out of 30 students (83.3%) did so with the conventional simulator. In the fourth attempt, 29 students (96.7%) achieved R0 resection with the in-house simulator, compared to only 22 out of 30 students (73.3%) with the conventional simulator. A graphical representation of the R0 resections for both simulators is displayed in Fig. 3. While the differences did not reach statistical significance, the corresponding effect size for attempt three and four were 0.302 and 0.327 (Cramers V) suggesting a potential trend.

### Video evaluation by two senior experts

**Fig. 4:**
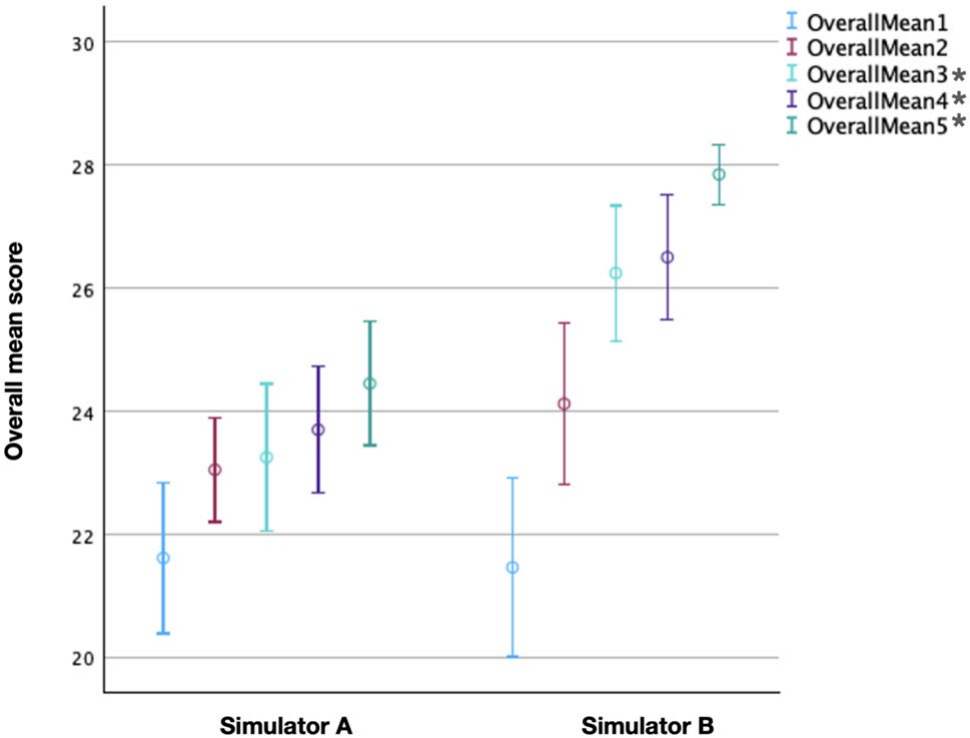
Overall mean score of simulator A and B over the course of five electrosurgical excisions. Dots represent the mean values. Vertical bars indicate the 95% confidence intervals (CI). Data points on the left correspond to simulator A (training with the conventional simulator), whereas those on the right represent simulator B (training with the in-house 3D-printed simulator). A statistically significant difference between the two simulators was found from attempt three onward

Initially, following the methodology of Wilson et al. [34], the interrater reliability between the two blinded senior experts was assessed using Cronbach α. This measure reflects the degree of consistency in the ratings provided by both assessors. Values above 0.75 are considered to represent very strong agreement. In our study, the inter-rater reliability was 0.70 for the checklist mean score, 0.80 for the GRS mean score, and 0.79 for the overall mean score. These results demonstrate strong consistency, confirming the validity of the data for further analysis. Subsequently, the mean scores for the checklist mean, GRS mean, and overall mean were compared between the two simulators across the five attempts. The checklist score assessed three key aspects, each rated on a scale from 0 to 3, with a maximum possible total of 9 points. The Global Rating Scale (GRS) evaluated four criteria, with each category scored between 0 and 5, allowing for a highest possible total of 20 points. To calculate the overall score, the checklist score and the GRS score were summed. Resulting in a maximum achievable score of 29. For the checklist mean, both simulators demonstrated an increase in mean scores. The mean score of the conventional simulator rose from 6.45 points in the first attempt to 7.50 points in the fifth attempt. Similarly, the in-house simulator showed an increase, starting at 5.75 in the first attempt and rising to 8.50 in the fifth attempt. The GRS mean values also showed a steady increase for both simulators over the five attempts. The conventional simulator's mean score increased from 15.17 in the first attempt to 16.95 in the fifth attempt. For the in-house simulator, the mean score started at 15.52 in the first attempt and increased to 19.20 in the fifth attempt. The overall mean values also steadily increased for both simulators. The mean score for the conventional simulator increased from 21.62 in the first attempt to 24.45 in the fifth attempt, while the in-house simulator’s mean score rose from 21.27 to 27.85. As with the LEEP score, the checklist mean, GRS mean, and overall mean for each attempt were compared between the two simulators using the Mann–Whitney-U-test. Effect size was calculated using Cohen’s d. The in-house simulator demonstrated a statistically significant advantage over the conventional simulator starting from the fourth attempt for the checklist mean, from the third attempt for the GRS mean and for the overall mean (Tables 2, 3, 4 and Fig. 4).

**Table 2:** Mean "checklist mean" scores across five electrosurgical excisions for both simulators

	LLETZ1	LLETZ2	LLETZ3	LLETZ4*	LLETZ5*
checklist mean (SD) (conventional)	6,45 (1,58)	7,15 (1,16)	7,10 (1,62)	7,15 (1,29)	7,50 (1,25)
checklist mean (SD) (in-house)	5,75 (1,68)	7,05 (1,53)	8,10 (1,16)	8,05 (1,08)	8,50 (0,72)
*p* value	0,083	0,944	0,009	0,006	< 0,001
*p* value adjusted (Holm)	0,414	> 0,999	0,066	0,051	0,005
Effect size (Cohen’s d)	− 0,460	− 0,018	0,711	0,753	0,990

^a^The checklist score evaluates three key aspects: (1) whether the procedure was completed in a single step, (2) whether an adequate tissue sample was obtained and (3) whether damage to the surrounding tissue was avoided. Each category is scored on a scale of 0 to 3 points, with a maximum possible total of 9 points

*indicate statistically significant differences between the conventional simulator and the in-house simulator

**Table 3:** Mean "GRS mean" scores across five electrosurgical excisions for both simulators

	LLETZ1	LLETZ2	LLETZ3*	LLETZ4*	LLETZ5*
GRS mean (conventional)	15,167	15,900	16,150	16,550	16,950
GRS mean (in-house)	15,517	16,967	18,083	18,500	19,200
*p* value	0,431	0,011	< 0,001	< 0,001	< 0,001
*p* value adjusted (Holm)	> 0,999	0,069	< 0,001	< 0,001	< 0,001
Effect size (Cohen’s d)	0,204	0,690	1,189	1,275	1,712

^b^The Global Rating Scale (GRS) score assesses four criteria: (1) tissue handling, (2) time and motion efficiency, (3) instrument handling, and (4) procedural fluidity. Each category was rated on a scale from 1 to 5 points, with higher scores indicating superior performance

*indicate statistically significant differences between the conventional simulator and the in-house simulator

**Table 4:** Mean "Overall mean" scores across five electrosurgical excisions for both simulators

	LLETZ1	LLETZ2	LLETZ3*	LLETZ4*	LLETZ5*
Overall mean (conventional)	21,617	23,050	23,250	23,700	24,450
Overall mean (in-house)	21,267	24,017	26,241	26,517	27,850
*p* value	0,700	0,083	< 0,001	< 0,001	< 0,001
*p* value adjusted (Holm)	> 0,999	0,414	0,003	0,002	< 0,001
Effect size (Cohen’s d)	− 0,100	0,459	1,072	1,111	1,588

^c^To calculate the overall score, the checklist score and the GRS score assigned by each expert is summed

*indicate statistically significant differences between the conventional simulator and the in-house simulator

### Students’ assessment of both simulators

A total of 60 participating students answered 21 identical questions for both simulators. Additionally, Group A answered one extra question, while Group B responded to two additional simulator-specific questions. Responses were recorded using a 10-point Likert scale (1: "strongly agree/very good" to 10: "strongly disagree/very bad"). In the following analyses, values are presented as mean with standard deviation (SD). The statement "The surgical simulation training was enjoyable" received a rating of 1.1 (0.43) in Group A and 1.1 (0.25) in Group B. Both cohorts indicated that the simulation training had a positive impact on their education. The statement "The simulation training improved my medical skills" was rated with 1.4 (0.56) in Group A and with 1.3 (0.64) in Group B. Similarly, "The simulation training enhanced my knowledge and medical skills in gynecological examinations" was rated with 1.56 (1.00) in Group A and with 1.23 (0.43) in Group B. Both groups stated that "Surgical simulation helps me in real-life surgeries", with scores of 1.53 (1.01) (Group A) and 1.37 (0.67) (Group B). Additionally, "I would like to participate in further simulation training in gynecology and obstetrics" was rated 1.3 (0.69) in Group A and 1.2 (0.68) in Group B. The broader statement "I would like to participate in simulation training in other medical specialties" received an average score of 1.3 (1.47 and 0.18) from both groups. Regarding professional interest, participants were asked whether "The training sparked or strengthened my interest in gynecology", with Group A rating it at 2.2 (1.25) and Group B at 2.0 (1.26). The statement "How well did the model simulate a LLETZ procedure?" was rated 2.4 (1.30) by Group A and 2.1 (0.78) by Group B. The question "Is the representation of an artificial cervical canal useful for surgical simulation?" received ratings of 1.3 (0.61) (Group A) and 1.2 (0.41) (Group B). Further, participants evaluated the variation between the cervix of a nulliparous vs. multiparous patient in surgical simulation. Those using the conventional simulator rated it 3.5(2.15), while the in-house simulator group rated it 2.6 (1.52). Students were also asked whether "Performing the LLETZ procedure enhanced their gynecological knowledge", with Group A rating it at 1.7 (1.09) and Group B at 1.4 (0.73). The question "Would you be able to perform a real LLETZ procedure under supervision?" was rated 3.3 (1.87) in Group B and 3.1 (1.39) in Group A. Participants were further surveyed on their confidence and knowledge regarding electrosurgery. When asked if they had "acquired sufficient technical knowledge about electrosurgery", Group A rated it 2.2 (1.32) and Group B 2.4 (1.25). Confidence in handling electrosurgery was rated at 2.3 (1.66) in Group A and 2.2 (1.02) in Group B. Regarding the improvement of operative skills through electrosurgery, Group A rated it 2.1 (1.54), while Group B gave it 1.8 (0.95) points. Participants using the conventional simulator were specifically asked, "I did not like working with raw meat", with 23% answering "yes" and 77% "no." For those using the in-house simulator, the variation in the length and depth of the artificial vagina for surgical simulation was rated 2.2 (1.37), while the ability to actively perform a Lugol iodine test scored an average rating of 1.6 (0.81). The only statistically significant difference was found in the question: "Training in electrosurgery improved my medical education" (*p* < 0.05), with Group A rating it 1.2 (0.38) and Group B 1.6 (0.93).

### Residents' assessment of both simulators

Overall, the simulator training received positive feedback. The statement "The operative simulation training was enjoyable" was rated 2.0 (1.41) by Group A and 1.0 (0.0) by Group B. The question "The simulation of surgical procedures helps me in real operations" received mean scores of 1.4 (0.55) (Group A) and 1.2 (0.45) (Group B). Furthermore, participants expressed a willingness to undergo additional simulation training in gynecology and obstetrics (Group A: 1.2 (0.45); Group B: 1.0 (0.0)). The statement "The simulation training improved my medical skills" was rated 3.2 (1.64) by Group A and 1.6 (0.90) by Group B. The assertion "The simulation training improved my knowledge and skills regarding gynecological examination" received scores of 3.6 (2.41) in Group A and 2.2 (1.80) in Group B. Regarding the simulation of the LLETZ procedure, participants indicated an improvement in gynecological knowledge (Group A: 3.6 (2.41); Group B: 1.2 (0.45)). The suitability of the simulators for replicating diagnostic procedures was also assessed. The simulation of a real PAP smear was rated 4.6 (3.78) by Group A and 2.0 (1.22) by Group B. The ability to simulate a cervical biopsy was rated 4.8 (3.90) (Group A) and 3.0 (2.35) (Group B). The replication of an endocervical curettage received scores of 5.4 (3.36) (Group A) and 3.6 (2.30) (Group B). The statement "Thanks to the simulator, I feel more confident in independently diagnosing cervical dysplasia" was rated 4.6 (2.3) in Group A and 1.6 (0.55) in Group B. Additionally, the potential of the simulator for LLETZ simulation was evaluated. The question "How well did the given simulator replicate a real LLETZ procedure?" was rated 4.0 (1.87) by Group A and 2.0 (0.71) by Group B. The artificial cervical canal representation received 1.2 points (0.45) in Group A and 1.0 points (0.0) in Group B. The inclusion of a variation between nulliparous and multiparous cervices was rated 2.8 (1.48) by Group A and 3.6 (2.07) by Group B. Regarding confidence in performing an actual LLETZ, Group A gave a mean of 4.4 (2.41) and Group B a mean of 1.6 points (0.55). Residents were also surveyed regarding their knowledge of electrosurgery. The statement "I have acquired sufficient technical knowledge in electrosurgery" received ratings of 3.0 1.87) (Group A) and 2.8 (1.10) (Group B). The assertion "Using electrosurgery has improved my surgical skills" was rated 3.0 (1.58) by Group A and 1.6 (0.90) by Group B. In the final overall assessment of electrosurgery training, Group B rated with 1.2 points (0.45) and Group A with 3.6 points (2.41). Furthermore, doctors who trained with Simulator A were asked if they found working with raw meat unpleasant. Three participants responded "no," while two answered "yes." The opportunity to actively perform a Lugol’s iodine test was rated 1.4 (0.89). The only statistically significant difference was found in the question: "I feel more confident handling electrosurgery" with Group A rating it 4.2 (1.64) and Group B 1.6 (0.89) (*p* = 0.032). Concerning the question "How well did the given model simulate a real LLETZ procedure?" Group A gave a mean of 4.0 (1.87) and Group B a mean of 2.0 (0.71) points (*p* = 0.056).

## Discussion

The in-house developed simulator demonstrated notable advantages over the conventional simulator across multiple performance indicators, suggesting its potential as a superior training tool for the LLETZ procedure. First, higher rates of R0 resections were achieved with the in-house simulator in the third and fourth training attempts (100% vs. 83.3 and 96.7% vs. 73.3). Second, to objectively assess surgical precision and learning progression, the LEEP score was employed. This score facilitated a standardized comparison between the simulators’ generated learning curve and revealed statistically superior results for the in-house simulator starting from the fifth attempt (adjusted *p* = 0.002). The effect size was large, with a Cohen’s d of 1.01. Third, these findings were confirmed by blinded video evaluations conducted by two senior clinicians using the evaluated, structured assessment tools "checklist mean” and “Global Rating Scale (GRS) mean,” which were combined into an overall mean score. The participants training with the in-house simulator received significantly higher ratings beginning from the fourth attempt in the checklist, and from the third attempt in the GRS and overall scores. These performance-based outcomes were further validated by participant feedback. Both students and residents reported a high degree of satisfaction with the training experience, emphasizing the simulator’s educational value and its impact on building confidence and skills in electrosurgery and LLETZ.

### Possible explanations for the superiority of the in-house simulator

Several factors may account for the superior performance observed with the in-house simulator. First, its wider vaginal opening allows for greater speculum expansion, enhancing visualization of the cervix and aceto-white lesions—an essential aspect for precise resections. While such visibility is often achievable in clinical practice, anatomical variability and patient discomfort can limit this. Given that the study focused on novices without prior experience, optimized and comfortable training conditions were a priority. Second, the consistency of the cervical model likely influenced performance. The in-house simulator uses agar–agar, whereas the commercial version employs processed meat. Prior research has highlighted the technical challenges posed by meat-based models, particularly regarding optimal loop excision speed [35]. Deviations in speed may lead to thermal damage or suboptimal excisions, potentially contributing to the variability observed in LEEP scores with the commercial simulator. Third, differences in material composition also affect smoke production during electrosurgery [36, 37]. Excessive smoke, more prominent in meat-based models, may impair visibility—especially for beginners—thus limiting the training’s effectiveness. Although lower smoke output in the in-house simulator may reduce perceived realism, it creates a more accessible learning environment. Taken together, the narrower vaginal canal, higher material-related demands, and increased smoke production likely rendered the commercial simulator more challenging. While such complexity could better simulate clinical reality, the primary objective of this study was to assess the suitability of simulators for training LLETZ novices—an aim more effectively met by the in-house simulator. More broadly, an important consideration in simulator-based training is whether differing levels of realism and procedural difficulty render certain models more suitable for specific stages of training. While simpler models may facilitate skill acquisition in novices, more complex simulators are often assumed to benefit advanced trainees. In our study, however, the in-house simulator was rated as at least equally realistic by participants with prior LLETZ experience, with a trend towards higher realism ratings, although this did not reach statistical significance. These findings do not support the concern that the in-house simulator may be “too easy” for more experienced users, but rather suggest that it provides an appropriate balance between usability and realism across different levels of training.

### Strengths and limitations

A notable strength of this study is the inclusion of both medical students and residents, thereby facilitating a comprehensive evaluation of simulator performance across varying levels of clinical experience. The cohort of medical students, all of whom lacked prior exposure to the LLETZ procedure, provided a homogeneous group with a uniform baseline of skills. This design minimized potential biases associated with differing levels of previous training or experience, which are often present among residents. The use of medical students in simulator evaluation is well-supported by existing literature, which attests to their suitability for such studies [35, 38, 39]. In addition, the inclusion of residents allowed for the validation of the students' perspectives, thus providing additional insights into the simulators realism and practical applicability. Another strength is the multi-dimensional assessment approach using established and validated metrics. The LEEP score, originally developed by Takacs et al. and previously employed by Kiesel et al. [30–34], along with the widely used OSATS framework (objective structured assessment of technical skills) [38, 40–44] offered reliable and comprehensive measures of performance. Furthermore, the in-house simulator demonstrated a substantial advantage in terms of cost efficiency. While the commercial simulator is priced at approximately EUR 1300 and requires additional consumables for each training session (cervical substitutes at approximately EUR 1 per unit), the in-house model can be produced at an estimated cost of EUR 263.11. Furthermore, the algae-based artificial cervix used in this model costs only around EUR 0.33 per unit. This considerable reduction in both initial and recurring costs makes the in-house simulator particularly well suited for repeated use in teaching settings and may facilitate broader accessibility in resource-limited training environments.

A limitation of this study is that no formal a priori power calculation was performed, as the sample size was determined pragmatically by the available teaching cohort. However, post hoc considerations suggest that, with 30 participants per group and a significance level of 0.05, the study was sufficiently powered (≈80%) to detect moderate-to-large effects (Cohen’s *d* ≈ 0.7–0.75). Even under more conservative assumptions (*α* ≈ 0.01), large effects (*d* ≈ 0.93) would remain detectable. As the observed effect sizes largely exceeded these thresholds, the sample size appears adequate for the primary analyses. Nevertheless, future studies with predefined power calculations and larger cohorts are warranted to strengthen generalizability. Another limitation is the relatively small sample size of residents, which reflects the challenges of recruiting participants with ongoing clinical responsibilities. The inclusion of a larger cohort and, ideally, an additional expert group with extensive procedural experience could further enhance the validity of the findings. The study also focused on short-term performance across five attempts and did not assess long-term skill retention or transferability to clinical practice. Consequently, conclusions regarding sustained competence remain limited. A further limitation is that the analysis was based on predefined attempts, particularly the final attempt, rather than modelling the full learning curve. This design was chosen to ensure comparability with the study by Takacs et al. [33], in which participants likewise performed five consecutive excisions. However, this approach does not allow identification of a potential mastery plateau. Future studies using longitudinal modelling and a higher number of repetitions may provide more detailed insights into skill acquisition dynamics. Finally, the internal consistency of the checklist was at the lower threshold of acceptability (Cronbach’s *α* = 0.70) and slightly lower than that of the GRS (*α* = 0.80). This difference is likely attributable to structural characteristics of the instruments rather than rater disagreement, as the checklist comprised only three items focusing on discrete procedural outcomes, whereas the GRS assessed broader aspects of technical performance. To account for these complementary properties, both scoring systems were combined into a composite mean score, enabling a more comprehensive assessment of performance.

### Transferability to real-life surgery and future aspects

Wilson et al. emphasized the need for additional research to better understand the correlation between simulator performance and real-world surgical competence [34]. A 2019 study demonstrated that simulation-based education led to improvements in specific aspects of procedural performance among trainees [44]. Several other studies have similarly supported the transferability of simulation skills to clinical practice [45–48]. However, this assumption has been questioned in other surgical fields. For instance, in colonoscopy, while simulator-trained physicians initially showed better performance, this advantage diminished after approximately 30 procedures, with no significant differences observed thereafter [49]. Despite this, early improvements in skill acquisition may optimize the use of instructor resources and enhance patient safety by elevating the baseline skill level of trainees. Given the relationship between the surgeon's experience and the risk of obstetric complications, an early and sustainable success of simulator training for LLETZ is desirable [8–14]. If the skills gained through simulation were entirely transferable, the in-house simulator—given its superior learning outcomes—would likely provide a more effective training experience. However, this remains a hypothetical assumption. Future research could explore whether participation in simulator-based training programs during residency results in improved outcomes in real-world surgical settings. Furthermore, integrating virtual reality (VR) technologies into simulator training represents another promising direction for future research. In other surgical disciplines, VR-based training has been shown to shorten learning curves [50] and it holds considerable potential for enhancing the effectiveness of LLETZ training as well.

## Supplementary Information

Below is the link to the electronic supplementary material.

Supplementary file1 (DOCX 28 KB)

## Data Availability

The datasets used and analysed during the current study are available from the corresponding author on reasonable request.

## Abbreviations

LLETZ: Large loop excision of the transformation zone
LEEP: Loop electrosurgical excision procedure
GRS: Global rating scale
VR: Virtual reality
OSATS: Objective structured assessment of technical skill
