# Supplementary material for: Comparative validation of a cost-effective in-house 3D-printed LLETZ simulator versus a commercial training model
Source: Arch Gynecol Obstet. 2026 Apr 4;313(1):151. doi: 10.1007/s00404-026-08413-3 (PMC13050339; doi:10.1007/s00404-026-08413-3)
Supplement: Supplementary file 1 — Supplementary file1 (DOCX 28 KB) [file 404_2026_8413_MOESM1_ESM.docx]

*Accociated date*

*Questionnaire – medical student – conventional simulator*

1. I enjoyed the surgery simulation training

1=strongly agree. 5=neutral. 10=strongly disagree

1. Have you ever perfomed a real Large Loop Excisions of the Transformation Zone (LLETZ) before

1=yes 2=no

1. How well could the current model simulate a LLETZ?

1=very good. 5=neutral. 10=very bad

1. How well could the current model simulate a Pap smear?

1=very good. 5=neutral. 10=very bad

1. How well could the current model simulate a biopsy of the cervix?

1=very good. 5=neutral. 10=very bad

1. How well could the current model simulate a curettage of the cervical canal?

1=very good. 5=neutral. 10=very bad

1. Is the illustration of an artificial endocervical canal helpful for surgical simulation?

1=very good. 5=neutral. 10=very bad

1. Is a variation between nulli- and multiparous cervical models helpful for surgical simulation?

1=very good. 5=neutral. 10=very bad

1. The simulation training has improved my medical expertise

1=strongly agree. 5=neutral. 10=strongly disagree

1. The simulation training has improved my knowledge and my medical expertise in gynecological examination

1=strongly agree. 5=neutral. 10=strongly disagree

1. I have received sufficient technical knowledge about electrosurgery

1=strongly agree. 5=neutral. 10=strongly disagree

1. I have gained self-confidence in the use of electrosurgery

1=strongly agree. 5=neutral. 10=strongly disagree

1. The application of LLETZ has improved my knowledge in gynecology

1=strongly agree. 5=neutral. 10=strongly disagree

1. As part of my general experience, electrosurgery training has imporved the quality of my medical training

1=strongly agree. 5=neutral. 10=strongly disagree

1. Simulation of surgery helps me later on dealing with real patients

1=strongly agree. 5=neutral. 10=strongly disagree

1. The application of electrosurgery improved my surgical skills

1=strongly agree. 5=neutral. 10=strongly disagree

1. I could do the the diagnosic procedure of cervical displasia (e.g. Pap smear, biopsy of the cevix, curettage of the cervical canal) under supervision myself

1=strongly agree. 5=neutral. 10=strongly disagree

1. I could perform a real LLETZ under supervision myself

1=strongly agree. 5=neutral. 10=strongly disagree

1. I wish to carry out more surgical simulation training in other clinical subjects

1=strongly agree. 5=neutral. 10=strongly disagree

1. I wish to carry out more simulation training in Obstetrics and Gynecology

1=strongly agree. 5=neutral. 10=strongly disagree

1. I disliked working with raw meat

1=yes 2=no

1. Working with the simulator increased my interest in gynecology.

1=strongly agree. 5=neutral. 10=strongly disagree

*Questionnaire – medical student – in-house simulator*

1. I enjoyed the surgery simulation training

1=strongly agree. 5=neutral. 10=strongly disagree

1. Have you ever perfomed a real Large Loop Excisions of the Transformation Zone (LLETZ) before

1=yes 2=no

1. How well could the current model simulate a LLETZ?

1=very good. 5=neutral. 10=very bad

1. How well could the current model simulate a Pap smear?

1=very good. 5=neutral. 10=very bad

1. How well could the current model simulate a biopsy of the cervix?

1=very good. 5=neutral. 10=very bad

1. How well could the current model simulate a curettage of the cervical canal?

1=very good. 5=neutral. 10=very bad

1. Is the illustration of an artificial endocervical canal helpful for surgical simulation?

1=very good. 5=neutral. 10=very bad

1. Is a variation in the artificial vagina's depth and width helpful for surgical simulation?

1=very good. 5=neutral. 10=very bad

1. Is a variation between nulli- and multiparous cervical models helpful for surgical simulation?

1=very good. 5=neutral. 10=very bad

1. Is the possibility of actively using Lugol's iodine helpful for simulation?

1=very good. 5=neutral. 10=very bad

1. The simulation training has improved my medical expertise

1=strongly agree. 5=neutral. 10=strongly disagree

1. The simulation training has improved my knowledge and my medical expertise in gynecological examination

1=strongly agree. 5=neutral. 10=strongly disagree

1. I have received sufficient technical knowledge about electrosurgery

1=strongly agree. 5=neutral. 10=strongly disagree

1. I have gained self-confidence in the use of electrosurgery

1=strongly agree. 5=neutral. 10=strongly disagree

1. The application of LLETZ has improved my knowledge in gynecology

1=strongly agree. 5=neutral. 10=strongly disagree

1. As part of my general experience, electrosurgery training has imporved the quality of my medical training

1=strongly agree. 5=neutral. 10=strongly disagree

1. Simulation of surgery helps me later on dealing with real patients

1=strongly agree. 5=neutral. 10=strongly disagree

1. The application of electrosurgery improved my surgical skills

1=strongly agree. 5=neutral. 10=strongly disagree

1. I could do the the diagnosic procedure of cervical displasia (e.g. Pap smear, biopsy of the cevix, curettage of the cervical canal) under supervision myself

1=strongly agree. 5=neutral. 10=strongly disagree

1. I could perform a real LLETZ under supervision myself

1=strongly agree. 5=neutral. 10=strongly disagree

1. I wish to carry out more surgical simulation training in other clinical subjects

1=strongly agree. 5=neutral. 10=strongly disagree

1. I wish to carry out more simulation training in Obstetrics and Gynecology

1=strongly agree. 5=neutral. 10=strongly disagree

1. Working with the simulator increased my interest in gynecology.

1=strongly agree. 5=neutral. 10=strongly disagree

*Questionnaire – residents – conventional simulator*

1. I enjoyed the surgery simulation training

1=strongly agree. 5=neutral. 10=strongly disagree

1. How well could the current model simulate a real LLETZ?

1=very good. 5=neutral. 10=very bad

1. How well could the current model simulate a Pap smear?

1=very good. 5=neutral. 10=very bad

1. How well could the current model simulate a biopsy of the cervix?

1=very good. 5=neutral. 10=very bad

1. How well could the current model simulate a curettage of the cervical canal?

1=very good. 5=neutral. 10=very bad

1. Is the illustration of an artificial endocervical canal helpful for surgical simulation?

1=very good. 5=neutral. 10=very bad

1. Is a variation between nulli- and multiparous cervical models helpful for surgical simulation?

1=very good. 5=neutral. 10=very bad

1. The simulation training has improved my medical expertise

1=strongly agree. 5=neutral. 10=strongly disagree

1. The simulation training has improved my knowledge and my medical expertise in gynecological examination

1=strongly agree. 5=neutral. 10=strongly disagree

1. I have received sufficient technical knowledge about electrosurgery

1=strongly agree. 5=neutral. 10=strongly disagree

1. I have gained self-confidence in the use of electrosurgery

1=strongly agree. 5=neutral. 10=strongly disagree

1. The application of LLETZ has improved my knowledge in gynecology

1=strongly agree. 5=neutral. 10=strongly disagree

1. As part of my general experience, electrosurgery training has imporved the quality of my medical training

1=strongly agree. 5=neutral. 10=strongly disagree

1. Simulation of surgery helps me later on dealing with real patients

1=strongly agree. 5=neutral. 10=strongly disagree

1. The application of electrosurgery improved my surgical skills

1=strongly agree. 5=neutral. 10=strongly disagree

1. Due to the simulator I feel more confident concerning the diagnosic procedure of cervical displasia (e.g. Pap smear, biopsy of the cevix, curettage of the cervical canal) myself.

1=strongly agree. 5=neutral. 10=strongly disagree

1. Due to the simulator I feel more confident performing a real LLETZ myself

1=strongly agree. 5=neutral. 10=strongly disagree

1. I wish to carry out more simulation training in Obstetrics and Gynecology

1=strongly agree. 5=neutral. 10=strongly disagree

1. I disliked working with raw meat

1=yes 2=no

*Questionnaire – residents – in-house simulator*

1. I enjoyed the surgery simulation training

1=strongly agree. 5=neutral. 10=strongly disagree

1. How well could the current model simulate a real LLETZ?

1=very good. 5=neutral. 10=very bad

1. How well could the current model simulate a Pap smear?

1=very good. 5=neutral. 10=very bad

1. How well could the current model simulate a biopsy of the cervix?

1=very good. 5=neutral. 10=very bad

1. How well could the current model simulate a curettage of the cervical canal?

1=very good. 5=neutral. 10=very bad

1. Is the illustration of an artificial endocervical canal helpful for surgical simulation?

1=very good. 5=neutral. 10=very bad

1. Is a variation in the artificial vagina's depth and width helpful for surgical simulation?

1=very good. 5=neutral. 10=very bad

1. Is a variation between nulli- and multiparous cervical models helpful for surgical simulation?

1=very good. 5=neutral. 10=very bad

1. Is the possibility of actively using Lugol's iodine helpful for simulation?

1=very good. 5=neutral. 10=very bad

1. The simulation training has improved my medical expertise

1=strongly agree. 5=neutral. 10=strongly disagree

1. The simulation training has improved my knowledge and my medical expertise in gynecological examination

1=strongly agree. 5=neutral. 10=strongly disagree

1. I have received sufficient technical knowledge about electrosurgery

1=strongly agree. 5=neutral. 10=strongly disagree

1. I have gained self-confidence in the use of electrosurgery

1=strongly agree. 5=neutral. 10=strongly disagree

1. The application of LLETZ has improved my knowledge in gynecology

1=strongly agree. 5=neutral. 10=strongly disagree

1. As part of my general experience, electrosurgery training has imporved the quality of my medical training

1=strongly agree. 5=neutral. 10=strongly disagree

1. Simulation of surgery helps me later on dealing with real patients

1=strongly agree. 5=neutral. 10=strongly disagree

1. The application of electrosurgery improved my surgical skills

1=strongly agree. 5=neutral. 10=strongly disagree

1. Due to the simulator I feel more confident concerning the diagnosic procedure of cervical displasia (e.g. Pap smear, biopsy of the cevix, curettage of the cervical canal) myself.

1=strongly agree. 5=neutral. 10=strongly disagree

1. Due to the simulator I feel more confident performing a real LLETZ myself

1=strongly agree. 5=neutral. 10=strongly disagree

1. I wish to carry out more simulation training in Obstetrics and Gynecology

1=strongly agree. 5=neutral. 10=strongly disagree
